# Changes in Resting-State Cerebral Activity in Patients with Hyperthyroidism: A Short-Term Follow-Up Functional MR Imaging Study

**DOI:** 10.1038/s41598-017-10747-7

**Published:** 2017-09-06

**Authors:** Bo Liu, Qian Ran, Daihong Liu, Si Zhang, Dong Zhang

**Affiliations:** 1 Department of Radiology, XinQiao Hosptial, Third Military Medical University, ChongQing, 400037 P.R. China; 2 Department of Radiology, Southwest Hosptial, Third Military Medical University, ChongQing, 400038 P.R. China

## Abstract

To investigate the brain functional abnormality of hyperthyroid patients before and after treatment for one month using resting-state functional magnetic resonance imaging (rs-fMRI). Amplitude of low-frequency fluctuation (ALFF) and seed-based functional connectivity (FC) analysis were performed in 27 new-onset untreated hyperthyroid patients relative to 30 healthy controls. In addition, follow-up data were available for 19 patients treated with methimazole for one month. Compared with healthy controls, patients exhibited lower ALFF in the right posterior cingulate cortex (PCC); increased FC in the bilateral anterior insula (AI), bilateral posterior insula (PI) and left anterior lobe of the cerebellum (ALC); and decreased FC in the bilateral lateral prefrontal cortex (LPFC), the right medial temporal gyrus (MTG) and the bilateral PCC. Compared with the hyperthyroid status, patients with improved thyroid function showed increased FC in the right LPFC and right dorsolateral prefrontal cortex (DLPFC). Subsequently, Pearson’s correlation analyses were performed between abnormal ALFF, FC, neuropsychological assessment and serum free triiodothyronine (FT3) levels. The results indicated that the alterations in regional and network-level brain functions, which might underlie different psychiatric complications were dynamic and interactional processes in hyperthyroidism. Moreover, the improvement in regional brain FC was correlated with the efficacy of anti-thyroid medication.

## Introduction

Dysfunction in thyroid hormones has been shown to exert a profound influence on emotional and cognitive deficits^1–3^. Hyperthyroidism, a common disease with the typical clinical feature of excessive circulating thyroid hormones, is frequently associated with a range of neuropsychiatric symptoms, including nervousness, irritability, depression, anxiety, memory impairment, poor concentration and tremulousness^4^. Previous studies have revealed that excessive thyroid hormones (THs), particularly free triiodothyronine (FT3), can induce oxidative stress and damage to neurons that modulate emotion and cognition^1, 5^. However, details of the latent mechanisms underlying this brain dysfunction and its reversibility remain to be elucidated.

Thyroid hormones are essential factor for brain development processes, such as neuronal differentiation, growth of neurospongium, synaptogenesis and dendritic proliferation^6, 7^. The parasecretion of circulating thyroid hormones in hyperthyroidism influences neural function in the regions of the brain with the highest concentrations of receptors^8^. The changes in cerebral structure and brain function induced by hyperthyroidism are currently being researched. One study using voxel-based morphometry (VBM) reported that the gray matter volume (GMV) in the bilateral hippocampus, parahippocampal gyrus and left temporal pole was decreased in hyperthyroid patients compared to controls, but the bilateral supplementary motor area was increased^9^. Moreover, in a group of 28 healthy volunteers with hyperthyroidism induced by taking 250 μg of tetraiodothyronine (T4) for 8 weeks, the GMV was increased in the right posterior cerebellum but was decreased in the bilateral visual cortex and anterior cerebellum compared to euthyroid patients^10^. A fluorodeoxyglucose (FDG) PET study reported that hyperthyroid patients exhibited lower activity in the limbic system, frontal lobes and temporal lobes relative to healthy controls. However, compared with the hyperthyroid status, anti-thyroid therapy (treatment with methimazole, mean 77 days) induced increased metabolism in the left parahippocampal, fusiform and right superior frontal gyrus^11^. There was a significantly decreased glutamate concentration in the posterior cingulate cortex of hyperthyroid patients relative to controls according to *in vivo* proton magnetic-resonance spectroscopy (^**1**^H-MRS)^12^. Resting-state functional magnetic resonance imaging (fMRI) analysis revealed that hyperthyroid patients showed weaker functional connectivity(FC) from the bilateral hippocampus to both the bilateral anterior cingulate cortex (ACC) and bilateral posterior cingulate cortex (PCC) than the controls, as well as decreased FC between the right hippocampus and right medial orbitofrontal cortex (mOFC)^13^. In 29 subjects with hyperthyroidism induced by 8 weeks of daily oral administration of 250 μg of levothyroxine, increased connectivity between the temporal lobe and cognitive control network was observed^14^. Li^15^ regarded abnormal degree centrality(DC) in the posterior lobe of the cerebellum (PLC) and medial frontal gyrus (MeFG) as seed regions to explore the aberrant FC of patients with hyperthyroidism. They reported that abnormal FC was related to several brain functional networks, such as the default mode network (DMN), attention network and cognitive network. Based on the abovementioned studies, we speculated that changes in brain function might underlie emotional and cognitive impairments in hyperthyroid patients. However, the correlation between anti-thyroid treatment and changes in brain function remains unclear.

In the present study, we applied resting-state fMRI to investigate the abnormalities of brain function in patients with hyperthyroidism before and after the intake of methimazole over a one-month period. More specifically, to measure differences in regional cerebral activity, the amplitude of low-frequency fluctuation (ALFF) approach was used to detect alterations of regional brain function in hyperthyroid patients compared with healthy controls. ALFF is thus a measure reflecting the spontaneous neural function of local brain regions^16^. ALFF analysis has been widely used to study baseline activity and to uncover the latent mechanism of neurodegenerative disease, such as Parkinson’s disease^17^ and schizophrenia^18^. To explore the aberrant covariation of the time-course activity across regional brains, the peak points of brain regions identified by ALFF analysis were used as seeds for whole brain FC analyses^19^. Compared with traditional seeding approaches to select particular brain regions with the prior hypothesis, this method of locating seeds could provide an unbiased study of abnormalities in full-brain function. Monitoring resting-state brain function might provide exhaustive information about regional activity and network-level cerebral function abnormalities pre-therapy and about regional and network changes with improved thyroid function. Based on the combination of ALFF and FC analyses, we sought to further investigate the mechanisms underlying emotional and cognitive impairments and their possible reversibility after anti-thyroid therapy over one month.

We hypothesized that changes in regional and network-level brain function in specific brain regions of hyperthyroid patients are correlated with neuropsychiatric evaluations, and some of these changes can be reversed with the improvement of thyroid hormones after anti-thyroid therapy.

## Results

### Demographic and clinical characteristics

There were no statistically significant differences in terms of age, gender, educational background or Mini-Mental State Examination (MMSE) or Montreal Cognitive Assessment (MoCA) scores between the 27 hyperthyroid patients and 30 healthy controls (Table 1). However, the hyperthyroid patients before anti-thyroid therapy showed significantly higher serum FT3 levels, State-Trait Anxiety Inventory (STAI) and Beck Depression Inventory (BDI) scores, alerting effect and executive effect in the Attention Network Task (ANT) and a lower accuracy rate in the Stroop and two-back tasks than the healthy controls. Serum free thyroxine (FT4) and thyroid-stimulating hormone (TSH) levels were not collected because these values were beyond the reference ranges in some patients.

**Table 1:** Demographic data, clinical data, mood and cognitive characteristics of the participants.

Characteristic	Healthy controls(n = 30)	Hyperthyroidism (n = 27)	Hyperthyroid patients (n = 19)		*p* Value	*p* Value
			Pre-therapy	Post-treatment		
Age (years)	30.46 ± 4.4	30.59 ± 7.87			0.942^**a**^	
Gender (male/female)	12/18	12/15			0.869^**b**^	
Education (years)	15.33 ± 1.35	14.81 ± 1.50			0.185^**a**^	
Total score on MoCA	26.43 ± 0.50	26.40 ± 0.50			0.846^**a**^	
Total score on MMSE	26.63 ± 0.66	26.81 ± 0.73			0.333^**a**^	
Disease duration(months)		6.92 ± 7.77	7.95 ± 8.64			
FT3 (pmol/l)	4.95 ± 1.02	35.43 ± 13.06	33.80 ± 13.21	9.38 ± 6.29	<0.001^**a**^	<0.001^**c**^
**Attention**						
ANT alerting effect (ms)	45.20 ± 18.93	63.96 ± 27.49	59.05 ± 27.78	38.79 ± 15.50	0.004^**a**^	0.003^**c**^
ANT orienting effect(ms)	37.56 ± 22.55	37.74 ± 20.24			0.976^**a**^	
ANT executive effect(ms)	94.03 ± 32.83	118.48 ± 40.18	114.47 ± 42.41	84.26 ± 34.7	0.014^**a**^	<0.001^**c**^
**Executive function**						
Accuracy of Stroop(%)	95.2 ± 3.7	90.70 ± 6.3	91.00 ± 7.06	94.95 ± 5.20	0.003^**a**^	<0.001^**c**^
Stroop RT(ms)	665.83 ± 84.81	747.44 ± 139.02	715.63 ± 129.48	609.73 ± 69.55	0.012^**a**^	<0.001^**c**^
**Work memory**						
Accuracy of two-back(%)	85.4 ± 6.4	74.5 ± 8.8	76.89 ± 8.9	86.00 ± 8.53	<0.001^**a**^	<0.001^**c**^
Two-back RT(ms))	683.26 ± 127.45	791.55 ± 149.88	747.57 ± 147.85	636.52 ± 96.08	0.005^**a**^	0.001^**c**^
**Emotion**						
SAI score	33.43 ± 8.94	54.37 ± 8.39	53.89 ± 7.44	37.15 ± 10.05	<0.001^**a**^	<0.001^**c**^
TAI score	34.1 ± 7.51	45.4 ± 12.98			<0.001^**a**^	
BDI score	4.03 ± 3.24	18.62 ± 12.26	20.31 ± 14.03	8.05 ± 7.44	<0.001^**a**^	0.001^**c**^

Abbreviations: MoCA = Montreal Cognitive Assessment; MMSE = Mini-Mental State Examination; FT3 = Free Triidothyronine; ANT = Attention Network Test; RT = Reaction Time; SAI = State Anxiety Inventory; TAI = Trait Anxiety Inventory; BDI = Beck Depression Inventory.

^a^Two independent-sample t-test (27patients *vs* 30 healthy controls).

^b^Chi-square test (27patients *vs* 30 healthy controls).

^c^Paired-samples t-test (pre-therapy *vs* post-treatment in 19 patients).

Compared with the hyperthyroid state, the 19 post-treatment patients showed a significant decrease in FT3, State Anxiety Inventory scores, BDI scores and the values of the alerting network and executive control network, but a significant increase was observed in the accuracy rate of the Stroop and two-back tasks. The abovementioned demographic and clinical characteristics of the subjects are reported in Table 1.

### ALFF analysis

**Figure 1:**
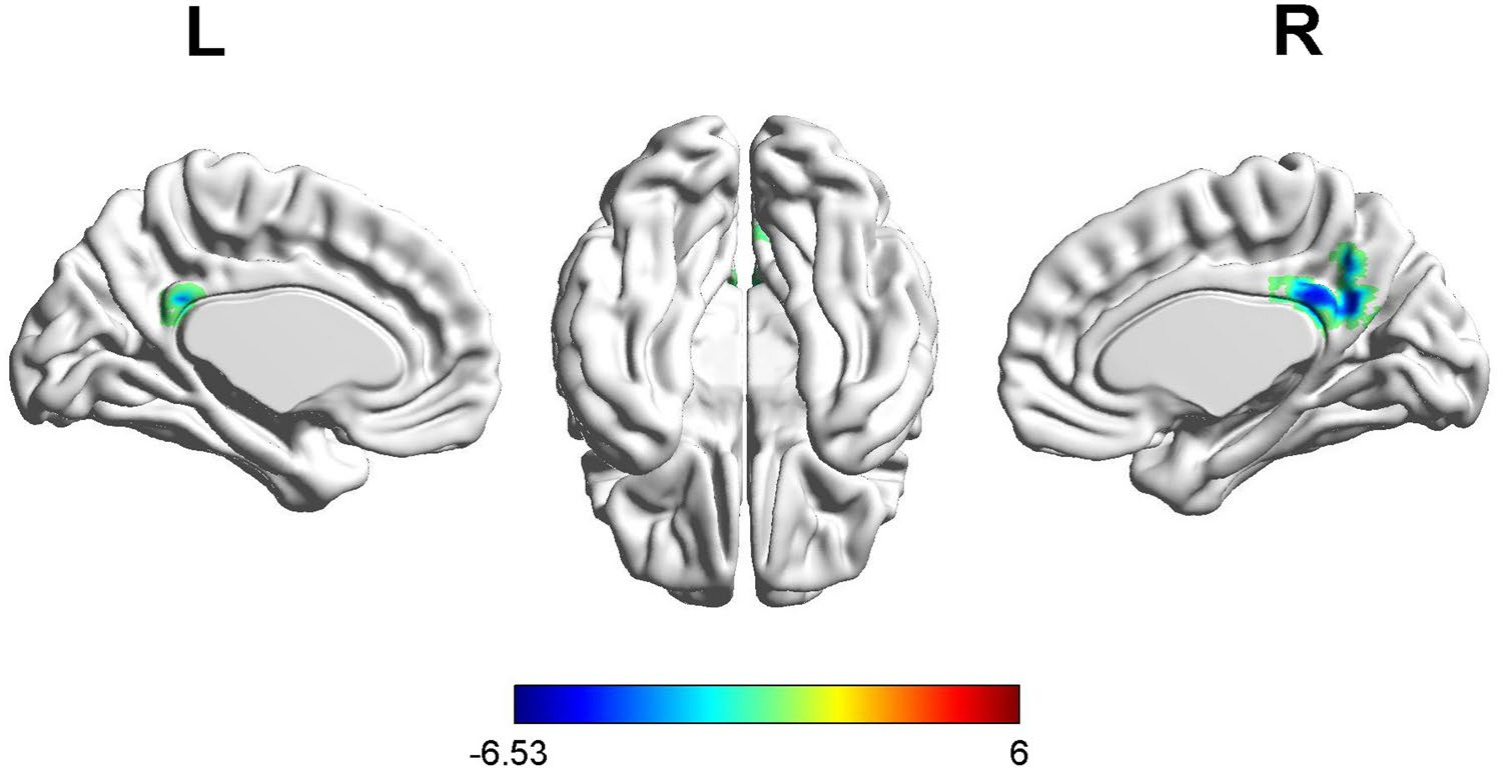
Significantly decreased (blue) ALFF in hyperthyroid patients compared with healthy controls (*p* < 0.001, AlphaSim-corrected). The color bar indicates the t-value from the two-sample t-test between the two groups.

Before treatment, the 27 patients showed a statistically significant ALFF decrease in the right PCC compared to the 30 healthy controls (*p* < 0.001, AlphaSim-corrected) (Table 2, Fig. 1). However, there was no significant ALFF difference after anti-thyroid treatment. The results are summarized in Table 2 (Fig. 1).

**Table 2:** Brain regions showing significantly different ALFF and FC between hyperthyroidism and control groups before and after anti-thyroid therapy (*p = *0.001, AlphaSim-corrected or small volume-corrected).

Brain region	BA	MNI coordinates			T-Value	Voxels
		X	Y	Z		
**ALFF** (pre-therapy, *p* = 0.001, AlphaSim-Corrected)						
Right PCC^**a**^	31_R	9	−60	27	−6.53	206
**FC** (pre-therapy, *p* = 0.001, uncorrected for multiple comparisons with a minimum cluster extent of 15 voxels)						
**Increased FC**						
Right PI^**a**^	48_R	39	−18	18	4.98	81
Left PI^**b**^	48_L	−39	−33	21	4.50	20
Right AI^**b**^	48_R	45	3	0	3.72	25
Left AI^**b**^	48_L	−30	3	15	3.80	20
Left ALC^**b**^		−15	−60	−27	3.89	19
**Decreased FC**						
Bilateral PCC^**a**^	29_R	9	−42	18	−5.28	210
Right LPFC^**a**^	10_R	15	57	0	−5.26	119
Left LPFC^**b**^	11_L	−18	54	−3	−4.44	28
Right MTG^**b**^	22_R	51	−51	21	−4.30	27
**FC** (post-treatment, *p* = 0.001, uncorrected for multiple comparisons with a minimum cluster extent of 15 voxels)						
**Increased FC**						
Right LPFC^**a**^	11_R	15	60	−9	5.07	52
Right DLPFC^**b**^	9_R	27	45	42	5.83	20

Abbreviations: MNI = Montreal Neurological Institute space; ALFF = Amplitude of Low-Frequency Fluctuation; BA = Brodmann area; PCC = Posterior Cingulate Cortex. FC = functional connectivity; PI = Posterior Insula; AI = Anterior Insula; ALC = Anterior Lobe of Cerebellum; PCC = Posterior Cingulate Cortex; LPFC = Lateral Prefrontal Cortex; MTG = Medial Temporal Gyrus; DLPFC = Dorsolateral Prefrontal Cortex.

^a^AlphaSim-corrected over the whole brain with significance at *p* < 0.001.

^b^Small volume-corrected (FWE-corrected at *p* < 0.05) with a cluster size threshold of 15 contiguous voxels.

### FC analysis

**Figure 2:**
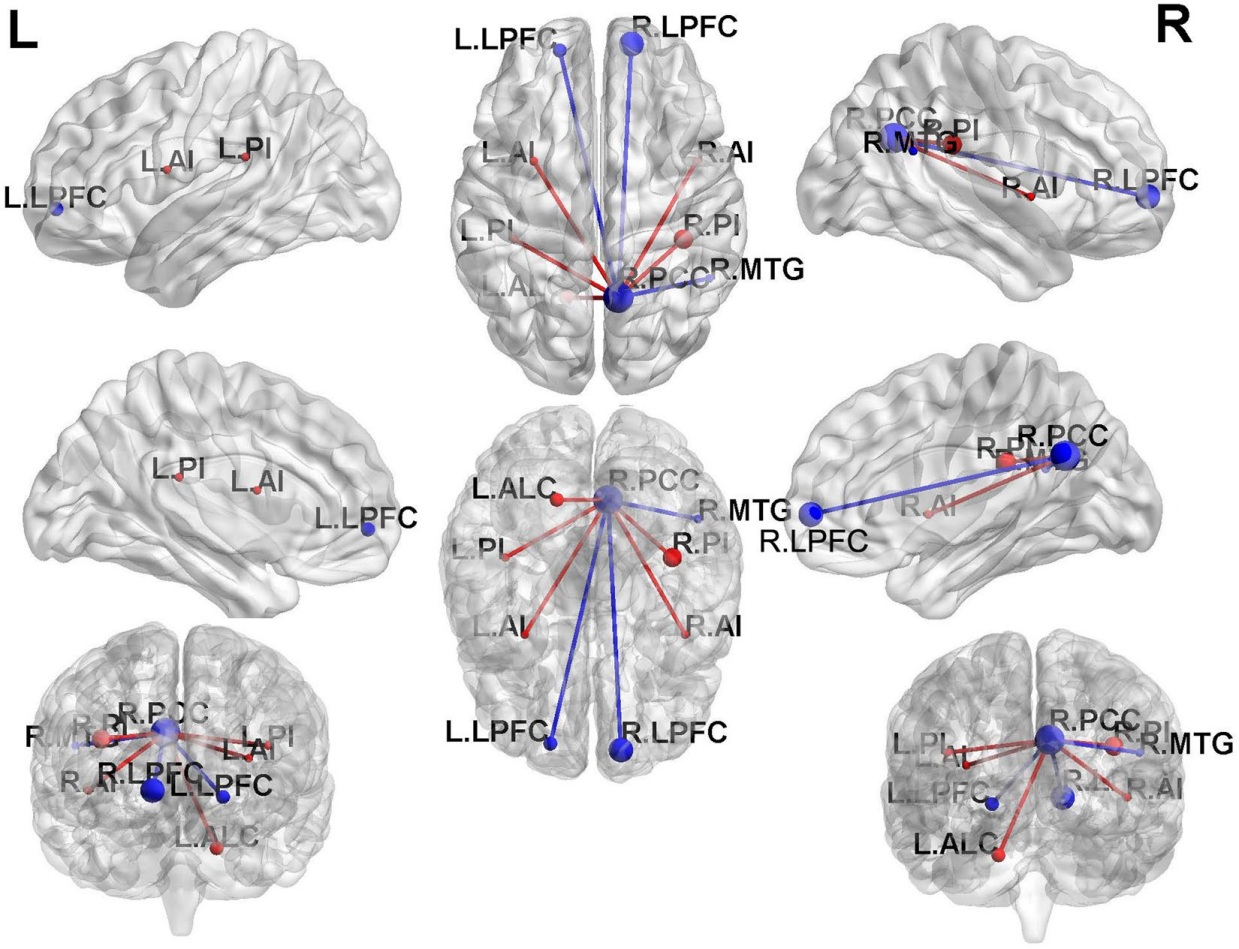
Differences in whole-brain FC between hyperthyroid patients and healthy controls before anti-thyroid therapy (*p* < 0.001, AlphaSim-corrected or small volume-corrected). The red color indicates increased FC, and the blue color means the decreased FC in the hyperthyroid group compared with the control group.

**Figure 3:**
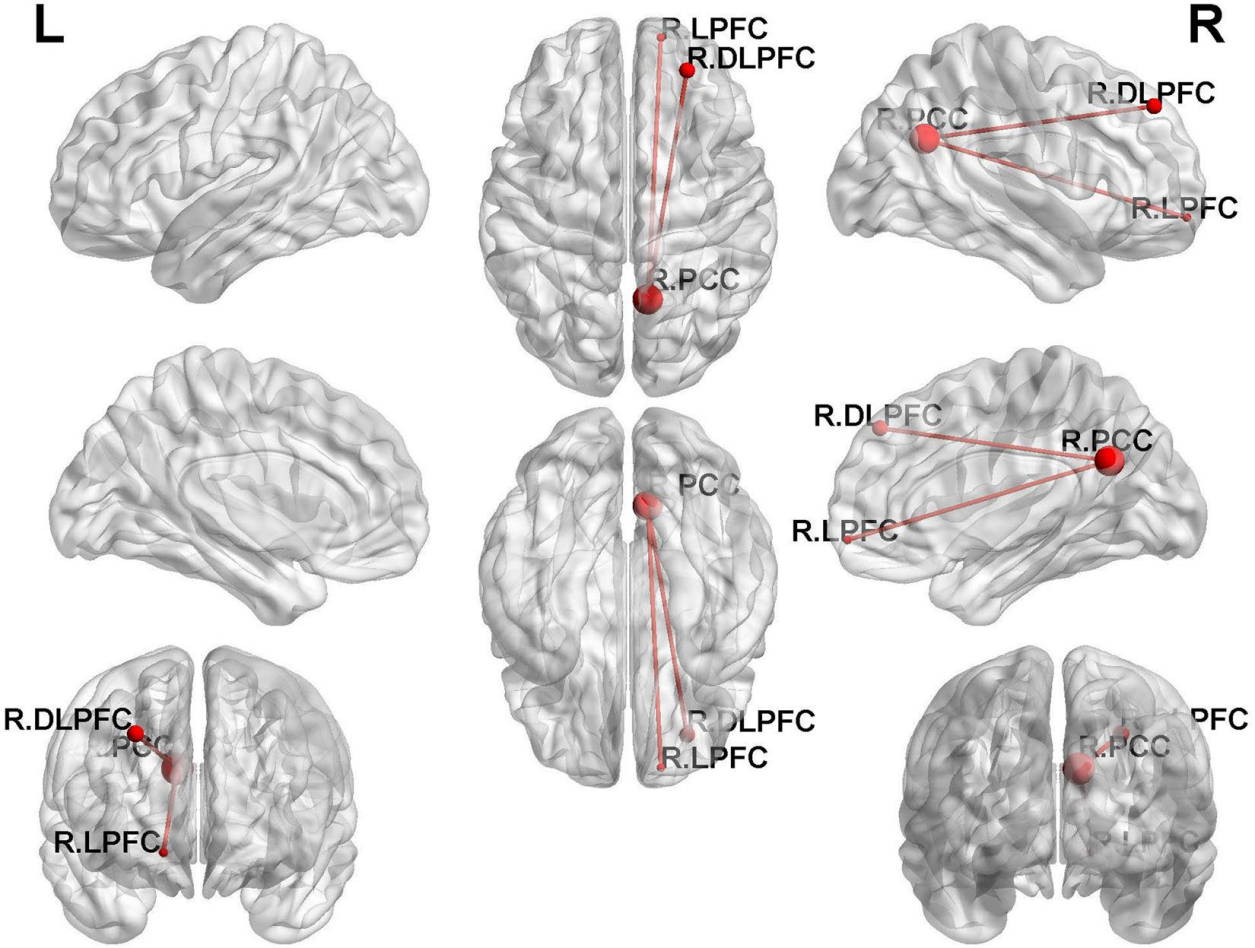
Differences in brain FC in 19 patients before and after one month of anti-thyroid therapy for one month (*p* < 0.001, AlphaSim-corrected or small volume-corrected). The red color indicates increased FC after anti-thyroid therapy compared with hyperthyroidism status.

Before treatment, the bilateral anterior insula(AI), posterior insula(PI) and left anterior lobe of the cerebellum(ALC) showed significantly stronger FC to the right PCC in the hyperthyroid group than the control group (*p < *0.001, AlphaSim-corrected or small volume-corrected) (Table 2, Figs 2 and 3). However, the bilateral LPFC, right medial temporal gyrus (MTG) and bilateral PCC showed significantly weaker FC from the right PCC in the hyperthyroid group than in healthy controls (*p* < 0.001, AlphaSim-corrected or small volume-corrected) (Table 2, Fig. 2). One month after treatment, significantly increased strength of FC between the right PCC and right LPFC and right DLPFC was found in the 19 hyperthyroid patients (*p* < 0.001, AlphaSim-corrected or small volume-corrected) (Table 2, Fig. 3).

### Correlation among clinical data, abnormal ALFF values and aberrant FC

**Figure 4:**
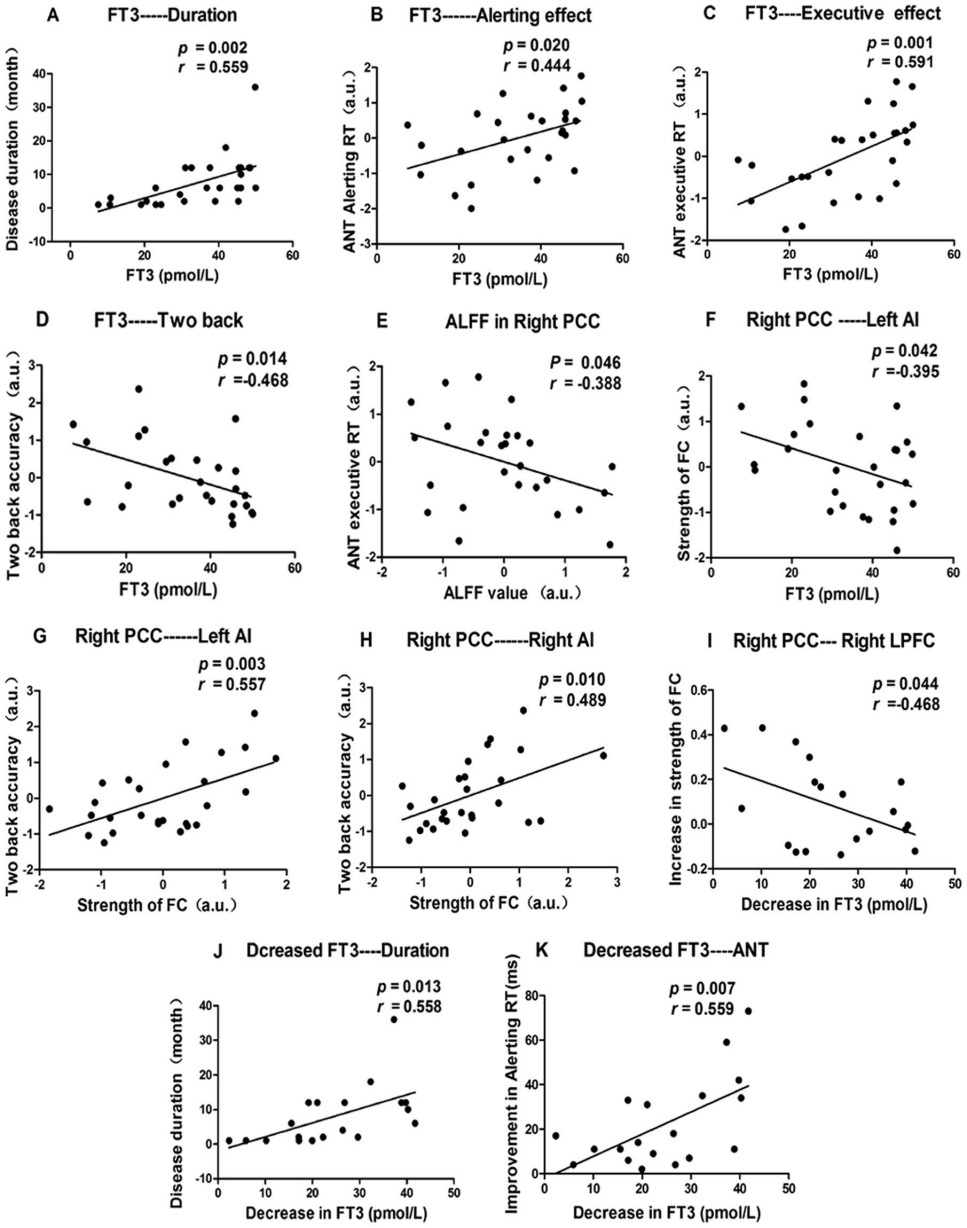
Scatter diagrams showing significant correlations between the clinical data, neurophysiologic assessment, values of ALFF and strength of FC in hyperthyroidism patients pre-therapy (**A–H**) and post-treatment (**I–K**). (**A**) FT3 levels were positively correlated with the duration of disease. (**B**) The alerting RT was positively correlated with FT3 levels. (**C**) The executive conflict RT was positively correlated with FT3 levels. (**D**) The accuracy of the two-back task was negatively correlated with FT3 levels. (**E**) The executive conflict RT was negatively correlated with the ALFF value in the right PCC. (**F**) The increased FC between the right PCC and left AI was negatively correlated with FT3 levels. (**G**) The accuracy of the two-back task was positively correlated with the increased FC between the right PCC and left AI. (**H**) The accuracy of the two-back task was positively correlated with the increased FC between the right PCC and left RI. (**I**) The increase in FC between the right PCC and right LPFC was negatively correlated with decrease FT3 levels. (**J**) The decrease in FT3 levels was positively correlated with the duration of disease. (**K**) The improvement in alerting RT was positively correlated with the decrease in FT3 levels.

Before anti-thyroid treatment, after controlling for the effects of age, sex and years of education, the results of Pearson’s correlation analysis yielded a positive correlation between FT3 levels and the duration of disease (*p* = 0.002, r = 0.559) (Fig. 4). Moreover, the ANT alerting effect and ANT executive effect were positively correlated with serum FT3 levels (*p* = 0.020, r = 0.444; *p* = 0.001, r = 0.591). The accuracy of the two-back task was negatively correlated with serum FT3 levels(*p* = 0.014, r = −0.468). Additionally, there was a negative correlation between ALFF values and the reaction time of the ANT executive effect (*p* = 0.046, r = −0.388). Finally, the increased strength of FC between the right PCC and left AI was negatively correlated with the serum FT3 levels (*p* = 0.042, r = −0.395), and the accuracy of the two-back task was positively correlated with the increased strength of FC between the right PCC and right AI (*p* = 0.010, r = 0.489) and left AI (*p* = 0.003, r = 0.557) (A–H in Fig. 4).

After the administration of anti-thyroid therapy, without controlling for the effect of age, sex or years of education, the magnitude of the increase in FC between the right PCC and right LPFC was negatively correlated with the decrease in FT3 levels (*p* = 0.044, r = −0.468). However, the decrease in FT3 was positively correlated with the duration of disease (*p* = 0.013, r = 0.558) and the improvement in the ANT alerting effect (*p* = 0.007, r = 0.559) **(**I–K in Fig. 4 **)**.

## Discussion

This investigation demonstrated brain function improvement in patients with hyperthyroidism after short-term treatment. We found the alterations of regional and network-level brain function in patients with hyperthyroidism. It should be noted that there was increased FC from the right PCC to the bilateral insula before treatment, and there was a negative correlation between the increase in the strength of FC from the right PCC to the right LPFC and the decrease in FT3 after anti-thyroid therapy. These findings suggest that the changes in regional and network-level brain function identified by rs-fMRI might be dynamic, and an interactive process underlies the neuropsychiatric manifestations of hyperthyroidism. Additionally, the change in FC was associated with therapeutic efficacy. This finding might suggest that these brain functional networks are correlated with the pathogenesis and progression of hyperthyroidism.

Before anti-thyroid therapy, hyperthyroid patients exhibited lower ALFF values in the right PCC and attenuated regional FC in the bilateral PCC. These results might indicate that the PCC has a vital role in brain regions consisting of DMN to regulate cognitive and mental functions in hyperthyroid patients. Based on the highest concentration of triiodothyronine (T3) receptors, excessive thyroid hormones could induce lower glucose metabolism in the PCC with increased disease severity^8^. The latent mechanism might be due to elevated THs that would disrupt the basal metabolism and change the respiratory rate in mitochondria to modulate reactive oxygen species (ROS) production. Additionally, THs regulate the neuronal antioxidant mechanisms in diverse pathways^5^. The imbalance in oxidative stress would cause considerably more damage in nerve corpuscles than in other tissues^20^. Previous studies also reported that patients with hyperthyroidism showed decreased glucose metabolism in the limbic system^8, 11^ and significantly decreased glutamate concentrations in the posterior cingulate cortex relative to healthy controls^12^. Aforementioned mechanism might account for the lower ALFF values of the right PCC in our study. The PCC is considered a cortical hub in the modulation of multimodal information^21^. Reduced metabolism in the PCC has been identified as an early sign of Alzheimer’s disease before a clinical diagnosis^21, 22^. The abnormal FC of DMN was involved in cognitive impairment^23, 24^ and emotional disorders^25, 26^. Previous investigations of untreated hyperthyroidism have revealed brain regions with disrupted FC anchored in DMN^13, 15^. Additionally, the decreased ALFF in the right PCC was negatively correlated with the reaction time on the ANT executive network. These results imply that the PCC plays a central role in the DMN to modulate the deterioration of neuropsychological performance in hyperthyroidism.

Moreover, the impairment of executive function in patients with hyperthyroidism might be associated with the abnormal FC between the bilateral LPFC and right PCC. Executive function is viewed as the fundamental ability of the LPFC. Patients with LPFC lesions have poorer performance on manipulation tasks, which require contextual decisions and supervising response-outcome relationships^27, 28^. Compared with healthy controls, patients with Graves’ disease showed significantly lower Cho/Cr ratios^29^ and decreased glucose metabolism in the frontal lobe^11^. The Iowa Gambling Task (IGT) study reported that patients with hyperthyroidism would rather choose option with a high immediate reward than choose a worse future punishment^30^. The current study suggest that the abnormal FC from the right PCC of the DMN to the bilateral LPFC may contribute to the cognitive disruption in executive function.

The increased FC from the right PCC to the bilateral insula(AI) might reveal the automatic regulation of the insula to the destruction of human homeostasis and the cognition of hyperthyroid patients. The insula plays roles in diverse functions, including interoceptive awareness^31^, motor control^32^ and cognitive functioning, which are usually involved in the regulation of the body’s homeostasis. Graves’ disease is anautoimmune thyroid disorder whose symptoms, including irritability, impulsiveness, bulimia and nervousness, are regulated by the insular cortex to maintain homeostasis^33^. As essential hub of the salience network (SN), the insula plays a critical role in cognitive control^34^ and in switching brain activity between introspective functions of the default-mode network and externally focused functions of the executive network^35^. Abnormalities in SN function would lead to dysfunction in cognitive control and may be a common nosogenesis underlying many psychiatric disorders^36^. Additionally, the positive correlation between the strength of FC from the right PCC to the bilateral AI and the accuracy of the two-back task might suggest that the regulation of the insula is beneficial for cognitive function. However, the negative correlation between FT3 and the strength of FC from the right PCC to left AI implied that the regulatory effect of the insula might weaken as hyperthyroidism worsens. Modulation of the insula may contribute to the common etiology of incipient cognitive impairment in patients with hyperthyroidism that are not overtly apparent.

In addition, considering the anterior cerebellum is polysynaptically connected to cortical motor areas, we suspected that the increased strength of FC between the right PCC and left ALC might contribute to modulating somatosensory symptoms, such as the tremor of the hands. Furthermore, the MTG is crucial for long-term memory^37^. Li^15^ reported that decreased FC between the right MTG and left PLC might result in cognitive impairment. Those results support the view that the reduced FC from the right PCC in DMN to the right MTG might be correlated with cognitive dysfunction.

Our results indicated that the dysfunction in regional FC after anti-thyroid treatment could be improved, and the improvement in FC was negatively correlated with the disease severity. A previous PET study demonstrated that treatment with methimazole induced a significant cluster of increased metabolic activity only in the right superior frontal cortex compared with the hyperthyroid status^11^. Another follow-up study with ^**1**^H-MRS also showed that the Cho/Cr ratios in the right prefrontal cortex were higher than pre-therapy^29^. In the current study, we speculated that the right frontal cortex may be a pivotal juncture in the restoration of brain function in patients with hyperthyroidism. In terms of the results of the correlation analysis, On the one hand, the positive correlation between the he decrease in FT3 and the duration of disease, which might suggest that when hyperthyroidism is more severe, the sensitivity to methimazole treatment is better. On the other hand, the recovery of the strength in FC from the right PCC to right LPFC was negatively correlated with the decrease in FT3, integrating the positive correlation between the decrease in FT3 and the duration of hyperthyroidism. This result might mean that when the severity of hyperthyroidism is more severe, the recovery of brain function occurs more slowly. Additionally, considering that a change in the DLPFC could not be found before anti-thyroid therapy, the reliability of this result should be further investigated.

Our findings extend previous studies by indicating that the right PCC might be a vital node in brain regions consisting of DMN to regulate cognitive and mental function as well as the automatic regulation of the insula. Moreover, the improvement in regional brain function was correlated with therapeutic efficacy in hyperthyroid patients. Zhang^13^ selected the bilateral hippocampus as a seed and reported decreased FC between the bilateral hippocampus and the ACC and PCC. The difference between the methods of locating seed regions might explain why we did not observe decreased FC in the ACC or bilateral hippocampus. Both Göttlich^14^ and Li^15^ used degree centrality (DC) and FC analyses to assess the changes in brain function in patients with hyperthyroidism. The former found increased DC in rostral temporal lobes and stronger FC from the temporal poles to the cognitive control network. The latter revealed reduced DC in the PLC and MeFG and observed that the abnormal FC was anchored in multiple brain networks. The crucial cause of this difference might lie in the subjects recruited in the two studies. Göttlich enrolled healthy men who received an 8-week regimen of daily oral administration of 250 μg of levothyroxine. However, the hyperthyroid subjects in Li’s study were all diagnosed with Graves’ disease and 15% percent of patients received treatment. However, hyperthyroidism is anautoimmune thyroid disorder with excessive FT3, FT4 and suppressed TSH. The thyrotoxicosis induced by the short-term oral administration of levothyroxine could not completely simulate its synthetic effect on patients. Finally, the treatment might have interfered with the results of brain function in Li’s research. Therefore, our investigation differs greatly from pre-existing research because we applied ALFF to patients with newly diagnosed and untreated hyperthyroidism and investigated the possible recovery of brain function after treatment.

There were several limitations in our study. First, the healthy controls did not receive the follow-up magnetic resonance imaging (MRI) scans. Considering that THs in hyperthyroidism would gradually return to normal after the three months of treatment according to clinical guidelines, the healthy controls were scheduled to receive MRI scans after 3 months in our follow-up experiments. Second, given the relatively small population (because it was limited to 19 post-treatment patients), and the absence of a a significant correlation when controlling for the effects of age, sex and years of education, the results of the post-treatment correlation analysis should be interpreted with caution, we would recruit more hyperthyroid patients to increase the population in follow-up experiment. Finally, compared with a clinical cure standard of no less than 12 months, the insufficient length of anti-thyroid therapy might have restricted the ability to investigate the treatment’s effect on brain function. This should be explored further until the thyroid function has completely recovered.

In conclusion, dysfunction in regional and network-level brain function might have a mixed effect on mood and cognitive disorders in hyperthyroidism. It was presumed that alterations in brain function were dynamic and interactional process occurring throughout the course of hyperthyroidism. The right PCC of DMN could be regarded as vital to aberrant brain function in patients with hyperthyroidism. Additionally, the improvement in regional cerebral function was associated with the efficacy of anti-thyroid therapy. Our findings might be helpful by providing information on the neural substrates supporting hyperthyroid-associated brain dysfunction and its possible reversibility with improved thyroid function.

## Methods

### Study design

The patients in the hyperthyroid group underwent serum thyroid hormone measurements, neuropsychological assessment and rs-fMRI scans before and one month after one month of methimazole therapy. During the treatment period, the dose of methimazole was modulated by the clinician according to the disease severity of each patient. For the healthy control group, all participants underwent the same comprehensive assessments at the beginning of the study, and they did not receive methimazole therapy.

### Subjects

The study was approved by the local Medical Research Ethics Committee of Xinqiao Hospital(Chong Qing, China), and the study was conducted in accordance with the approved guidelines and regulations. All participants provided their written informed consent after receiving a detailed description of the study procedures and aims. The experimental group recruited 27 right-handed patients (15 females and 12 males, age range: 18–50 years, mean age: 30.59 ± 7.87 years) with newly diagnosed and untreated Graves’ hyperthyroidism from the Endocrinology Department of XinQiao Hospital. All patients had elevated FT3 and FT4 and depressed TSH. The onset of clinical symptoms, including neuroticism, erethism, tremor, bulimia, easy fatigability and rapid emaciation, was recorded to determine the duration of hyperthyroidism. One month after anti-thyroid treatment, only 19 patients with hyperthyroidism were recalled to undergo the second comprehensive assessment. The control group consisted of 30 healthy right-handed volunteers(18 females and 12 males, age range: 18–50 years, mean age: 30.46 ± 4.4 years) who were recruited by networking in the local community. The same tests of thyroid function were administered to the control subjects, and all TH levels were within normal ranges (FT3 = 3.1–6.89 pmol/l, FT4 = 11.0–22.0 pmol/l, TSH = 0.27–4.2 mIU/ml).

The following exclusion criteria were applied to all subjects: a history of head injury, psychiatric disorders, alcohol or drug abuse, serious physical illness and contraindications to MR scanning. Two experienced neuroradiologists inspected the T_**1**_MR images to exclude gross neuroanatomic abnormalities. The final study groups contained 27 hyperthyroid patients before and 19 patients after receiving methimazole therapy as well as 30 healthy controls. The healthy control group was well matched with the untreated hyperthyroidism group according to sex, age and years of education.

### Neuropsychological assessment

Every subject underwent a series of neuropsychological assessments to evaluate variations in different cognitive territories at the beginning of the study. All patients with hyperthyroidism underwent a second neuropsychological assessment after anti-thyroid treatment for one month. Cognitive functions were assessed in all subjects using the MoCA and MMSE at the beginning of the study. The STAI is an introspective psychological inventory consisting of 40 self-reported items to assess anxiety symptoms^38^. The severity of depressive symptoms was measured by the Beck Depression Inventory II, containing a 21-question multiple-choice self-report inventory^39^.

The ANT provided measures of three independent attention networks (alerting, orienting and executive conflict) within a single task^40^. We calculated the “attention network efficiencies” according to the differences in reaction time (RT) under different type of cues and flanker stimuli. To assess the cognitive control function of patients with hyperthyroidism, the Stroop Color Word Test and a two-back working memory task were applied for all participants. The evaluation of the test results considered the accuracy of answers and the mean reaction time during the task.

### MRI data acquisition

Every participant underwent rs-fMRI scanning at the beginning of the study, and all patients underwent a second scan after one month of anti-thyroid treatment. During the process of scanning, the participants were required to remain equanimous, keep their eyes closed, stay awake and systematically think of nothing. Structural and functional MRI images were acquired using a 3.0 T GE MRI system equipped with a standard 8-channel head coil. Functional images were recorded axially by an Echo Planar Imaging (EPI) sequence using the following parameters: 34 slices, slice thickness = 5 mm and no slice gap, TE = 30ms, TR = 2300 ms, flip angle (FA) = 90°, field of view (FOV) = 240 × 240 mm^2^, matrix = 64 × 64 and isotropic voxel size = 3 × 3 × 3 mm³. For each subject, fMRI scanning lasted 620 s, and 270 volumes were obtained. High-resolution structural images were collected applying by a three-dimensional fast spoiled gradient-echo (3DSPGR) sequence with the following parameters: 124 slices, slice thickness = 1.6 mm and no slice gap, TE = 2.8ms, TR = 450ms, FA = 15°, FOV = 240 × 240 mm², matrix = 256 × 256 and isotropic voxel size = 1.6 × 1.6 × 1.6 mm³.

### MRI data analysis

The MRI data were preprocessed with the Data Processing & Analysis for (Resting-State) Brain Imaging(DPABI)^41^ (http://rfmri.org/dpabi) on the MATLAB 7.14.0(R2012a) platform. The first 10 volumes were deleted because of magnetization equilibrium and participants’ adaptation to the scanning environment. The remaining images were slice-time corrected and realigned for head motion. No images were removed from the results due to head motion >3.0 mm in any direction of x, y and z or 3.0°of any angular dimension. Next, the high-resolution T_1_-weighted images were used to coregister the functional images. Subsequently, a regression of nuisance variables, including average signals from cerebrospinal fluid, white matter, Friston 24-parameter correction and head motion scrubbing, was performed. In particular, because the removal of global brain signals remains controversial, we chose to retain the global signals. Then, all the data were filtered at the 0.01–0.08 Hz band and normalized to the Montreal Neurological Institute (MNI) template. Finally, smoothing with a Gaussian kernel of 6-mm full-width at half-maximum (FWHM) was performed, and detrending was applied to remove linear trends.

ALFF calculations were performed using the preprocessed images with temporal bandpass filtering (0.01 < f < 0.08 Hz) to reduce low-frequency drift and high-frequency respiratory and cardiac noise.

The calculation of FC was performed with rs-fMRI data analysis toolkits^42^ (REST v1.8) (http://www.restfmri.net/forum/REST_V1.8). The coordinates of the seed regions were defined according to the results of the ALFF analysis. The radius was 6 mm, and connectivity maps were calculated between the time courses of seed regions and the time series of all voxels in the global brain. Finally, Fisher’s *r*-to-*z* transformation was applied to all maps of ALFF and FC before the statistical analysis.

### Statistical analysis

Differences in demographic and clinical data between the 27 patients and 30 healthy controls were tested using a two-sample t-test with SPSS 20.0 for Windows(SPSS, Chicago, IL, USA). The Chi-square test was used to detect differences in sex. Next, the paired-samples t-test was used to evaluate the effects of anti-thyroid therapy on clinical data in the group of 19 patients. Significance was set at *p* < 0.05.

ALFF and FC map analyses were performed with SPM8 software (http://www.fil.ion.ucl.ac.uk/spm/). To characterize the pretherapeutic differences in ALFF and FC in the 27 hyperthyroid patients compared with the 30 normal subjects, a two-sample t-test was performed voxel-by-voxel after controlling for several covariates, including age, sex, mean value of head motion and years of education. Then, a paired-samples t-test was used to assess the possible changes in ALFF and FC after the administration of methimazole for one month in the group of 19 patients. Head motion was used as a covariate. For all of the above analyses, significance in the resulting statistical maps was set at 0.001, and a gray matter (GM) group mask was involved in the ALFF and FC calculations. The correction of results for multiple comparisons was divided into two parts: initially, to precisely define the peak points of brain regions with abnormal local brain activity in hyperthyroid patients as the seed regions for further FC analysis. The result maps of ALFF were corrected with the AlphaSim program (individual voxel *p*-value = 0.001, GM group mask, iteration = 1000) in DPABI software. The statistical threshold for pre-therapy ALFF was set at *p* = 0.001 and cluster size >35 voxels. Subsequently, the primary results of FC were displayed as statistical parametric maps (SPMs) in a standard MNI space at an initial threshold probability of *p* = 0.001 (uncorrected for multiple comparisons) and a cluster size threshold of 15 contiguous voxels. To fully explore the possible changes in brain connectivity before and after anti-thyroid therapy, statistical inference was performed in two steps with regard to the results of whole-brain FC calculations. First, a relatively strict statistical threshold of *p* = 0.001 in AlphaSim corrected for multiple comparisons was applied to the whole brain. The statistical threshold for FC was set at *p* = 0.001 and cluster size >68 voxels in pre-therapy and cluster size >44 voxels in post-treatment. Second, based on the results reported in previous studies^10–15, 29^ for the posterior cingulate cortex, frontal cortex, anterior cingulate cortex, temporal gyrus, cerebellum, insula and hippocampus, we used small volume-corrected (SVC) over these brain regions predicted *a priori* to demonstrate abnormalities in hyperthyroidism. These flexible statistical analyses were conducted using a family-wise error(FWE)-corrected threshold of *p* < 0.05 over the volume of the SVC-based hypothetical region, with a cluster size threshold of 15 contiguous voxels. Compared with a stricter correction for the whole brain, the SVC method enables hypothesis-driven analyses to be conducted with correction for multiple comparisons particularly in the cerebral region of interest^43, 44^.

To determine the relationships among clinical measures, the ALFF values and the strength of FC in the hyperthyroid patients, Pearson’s correlation analyses were performed with SPSS software after controlling for the effects of age, sex and education level, and the statistical threshold was set at *p* < 0.05.
